# The effect of antithrombotic therapy on the recurrence and outcome of chronic subdural hematoma after burr-hole craniostomy in a population-based cohort

**DOI:** 10.1007/s00701-022-05337-0

**Published:** 2022-08-16

**Authors:** Santtu Kerttula, Jukka Huttunen, Ville Leinonen, Olli-Pekka Kämäräinen, Nils Danner

**Affiliations:** 1 Institute of Clinical Medicine – Neurosurgery, University of Eastern Finland, Kuopio, Finland; 2 Neurocenter – Neurosurgery, Kuopio University Hospital, Kuopio, Finland

**Keywords:** Chronic subdural hematoma, Antithrombotic therapy, Outcome, Recurrence, Mortality, Bilateral chronic subdural hematoma

## Abstract

**Purpose:**

To study the effect of antithrombotic therapy (ATT) on the outcome of operatively treated chronic subdural hematomas (CSDH).

**Methods:**

A retrospective population-based cohort study from Eastern Finland including all adult patients who underwent a burr-hole craniostomy (BHC) for CSDH during 2016 and 2017. The follow-up time for recurrence was 6 months and for mortality 3 years.

**Results:**

A total of 301 CSDH patients were included in the study. ATT (antithrombotic therapy; antiplatelet or anticoagulant medication) was used by 164 patients (54.5%) at the time of diagnosis. The hematoma was bilateral in 102 patients (33.9%). Forty-seven patients (15.8%) encountered hematoma recurrence. Bilateral CSDHs required reoperations more often than unilateral hematomas (12.6% vs. 22.0%; *p* = 0.036) regardless of the primary operation (uni- or bilateral). A bivariate logistic regression analysis showed that bilateral hematoma (OR 1.918; 95% CI 1.013–3.630; *p* = 0.045) and male gender (OR 2.363; 95% CI 1.089–5.128; *p* = 0.030) independently predicted hematoma recurrence. The overall three-year mortality was 27.9%. The use of ATT was not associated with CSDH recurrence, and the length of the temporary postoperative ATT discontinuation did not correlate with the rate of thromboembolic events.

**Conclusions:**

ATT did not affect CSDH recurrence in our study population, and the duration of the temporary postoperative ATT discontinuation was not associated with the rate of thromboembolic complications. Male gender and bilateral hematomas were more frequently associated with recurrences.

## Introduction

Chronic subdural hematoma (CSDH) is a frequently encountered entity in the aging population. The overall incidence in adults ranges widely between 1.7 and 20.6/100,000/year [53], and in the age group of ≥ 80 years an annual incidence of up to 129.5/100,000 has been reported [40]. CSDH is defined as a pathological, slowly growing encapsuled collection of fluid in the subdural space [31]. It often originates from a preceding head trauma with a certain time interval but the trauma may be mild or even absent [6, 39]. In addition to a head trauma, age, male gender, alcohol abuse and antithrombotic therapy (ATT) with either anticoagulant or antiplatelet medications are perceived as risk factors for CSDH. [19, 31, 40] The pathophysiology of CSDH is attributed to a gradual inflammatory process induced by an initially small hematoma, which leads to fluid accumulation in the subdural space and progressive growth [16]. Surgical treatment is recommended for symptomatic CSDHs, and multiple different operative techniques have been suggested [2, 15, 25, 51]. Most operations can be performed under local anesthesia, which enables surgical treatment even for frail and comorbid patients. Among these patient groups ATT is commonly used, but the effect on the outcome of CSDH is controversial as is the risk of thromboembolic events related to temporarily discontinuing these medications. In this population-based study, we retrospectively collected all patients from Eastern Finland who underwent burr-hole craniostomy (BHC) for CSDH at Kuopio University Hospital during years 2016 and 2017. The aim was to study the effect of ATT on the outcome of operatively treated CSDHs and to identify risk factors for recurrence.

## Materials and methods

Kuopio University Hospital (KUH) is responsible for providing all neurosurgical treatment for its geographically defined catchment population (813,000 in 2016) in Eastern Finland. In this population-based cohort study, we identified all patients who were operated due to CSDH at KUH during the years 2016 and 2017.

**Fig. 1:**
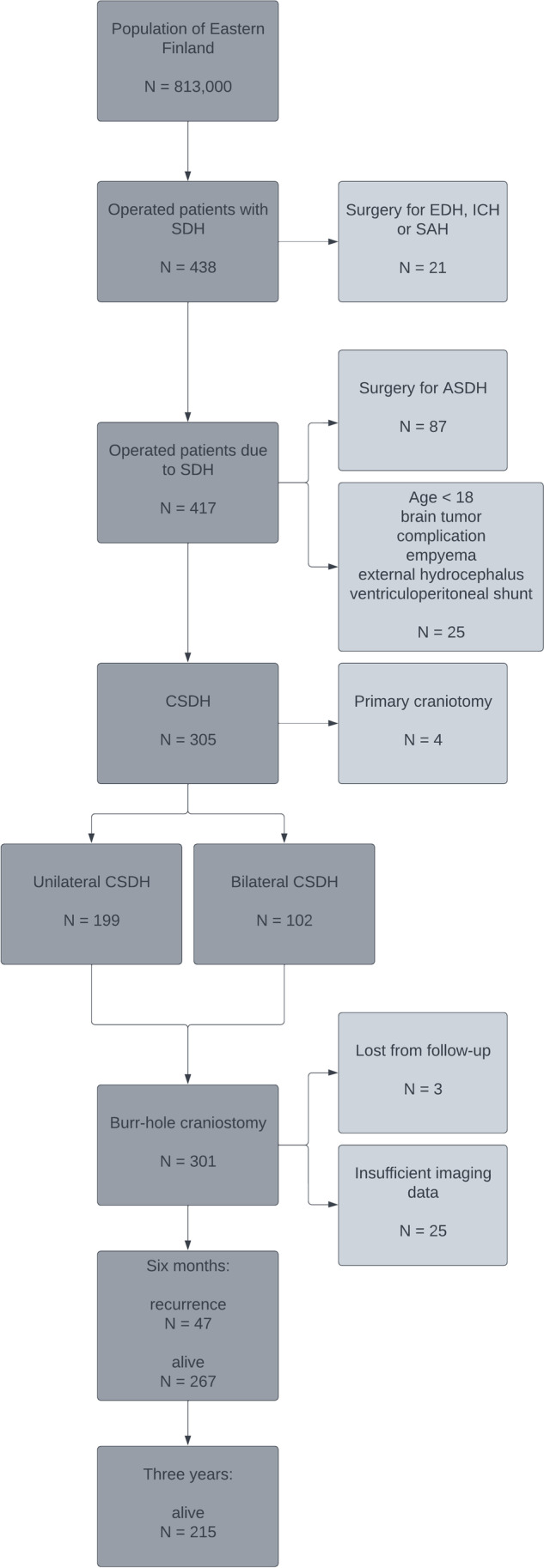
The flow chart of the study: Legends: EDH epidural hematoma, ICH intracerebral hemorrhage, SAH subaracnoid hemorrhage, ASDH acute subdural hematoma. (Lucidchart online diagramming application was used to create this figure)

The flow chart of the study is presented in Fig. 1. The final study population consisted of 301 patients. The medical records of all patients were reviewed in detail. In addition to recording demographic and clinical data, several grading scales were reconstructed from the details provided in the patient records. Data on antithrombotic medications were gathered from patient records and national prescription registries. The etiology of the hematoma was considered non-traumatic if no evidence of preceding head trauma, fall or other traumatic event was present in the medical records. Risks for bleeding and thromboembolic events were evaluated by calculating the HAS-BLED [36] and CHA_2_DS_2_-VASc [30] scores also for patients without atrial fibrillation diagnosis. HAS-BLED ≥ 3 points indicates high bleeding risk, and CHA_2_DS_2_-VASc ≥ 2 points indicates a high risk for thromboembolic events [29]. The Glasgow Coma Scale (GCS) [47] and the Markwalder Grading Scale (MGS) [31] were used to classify the severity of symptoms on admission. The Modified Rankin Scale (mRS)[46] was assessed before surgery and at the latest follow-up.

All patients underwent a single burr-hole craniostomy (uni- or bilateral) with intraoperative saline irrigation followed by the insertion of a closed subdural drainage system. A routine control CT scan was performed 1 month after surgery unless clinical deterioration indicated earlier imaging. The routine radiological follow-up was prolonged beyond 1 month on an individual basis if warranted by a clinically significant residual hematoma. Hematoma recurrence was defined as a radiologically confirmed symptomatic CSDH in the same location as the primary hematoma requiring a new operation within 6 months of the primary operation. If a bilateral hematoma was primarily treated with unilateral BHC, a contralateral-sided BHC was also counted as a reoperation. The follow-up was organized by the treating neurosurgical center. After the temporary discontinuation of ATT, the permission to resume the medication was also given by the same neurosurgical unit. Recurrence data was recorded until 6 months after the primary operation and mortality until 3 years.

### Statistical analyses

The data was analyzed using IBM SPSS Statistics software (version 27.0.1.0). The level of statistical significance was set to 0.05. Statistically significant differences (*p* < 0.05) between groups were calculated using Pearson’s Chi-square test for nominal variables unless the subgroup sample was small (*n* < 30), in which case Fisher’s exact test was used. Independent samples *t* test was used for comparing means, but for small (*n* < 30) subgroups we used independent samples Mann–Whitney *U* test. A bivariate logistic regression analysis was performed with an enter method using factors which had shown statistically significant difference between patient groups. Adjusted odd ratios (OR) with their 95 percent confidence intervals (CI) were computed. Kaplan–Meier analyses were performed to visualize 3-year survival and 6-month recurrence, but patients who died before the 6-month follow-up were excluded from the latter analysis, unless they experienced hematoma recurrence prior to their death.

## Results

A total of 301 patients were included in the final study cohort (Fig. 1). The mean age was 76.6 (± 10.4) years, and the proportion of male patients was 200/301 (66.4%). ATT was used by 164 patients (54.5%) at the time of diagnosis, the largest drug subgroups being warfarin (95 patients) and low-dose ASA (56 patients). Direct oral anticoagulant medication was used by four patients (rivaroxaban or dabigatran) and other antiplatelet therapy (clopidogrel, ticagrelor or a combination of low-dose ASA and dipyridamole) by twenty-one patients. Ten patients were on simultaneous warfarin and antiplatelet therapy. A preceding traumatic event was noted in 203 patients (67.4%). Bilateral hematomas were found in 102 patients (33.9%), and of those thirty-nine (38.2%) were primarily operated with bilateral BHC. The overall 3-year mortality was 27.9% (83 of 298 patients).

Forty-seven patients (15.8%) encountered hematoma recurrence which demanded operative treatment. The characteristics of patients with and without hematoma recurrence are compared in Table 1. ATT was not associated with hematoma recurrence (54.2% vs. 55.3%; *p* = 0.886). The mean time from primary surgery to reoperation was shorter for patients with ATT as compared to patients without ATT, but the difference was not statistically significant (43.0 days vs. 54.1 days; *p* = 0.323). The mean time from primary surgery to the resumption of medication was significantly longer in patients who experienced a recurrence as compared to patients who required only one operation (59.7 days vs. 122.0 days; *p* < 0.001), but there was no difference in the incidence of thromboembolic events between the groups. The HAS-BLED score was not associated with recurrence, and the CHA_2_DS_2_-VASc score did not predict thromboembolic events.

**Table 1:** Comparison of patients with and without hematoma recurrence

	No recurrence *N* = 251	Recurrence *N* = 47	*p* value
Age (years, mean ± SD)	76.5 ± 10.7	77.9 ± 8.6	0.396
Gender (male, *N* (%))	160 (63.7%)	38 (80.9%)	0.023^*^
Memory disorder	55 (21.9%)	12 (25.5%)	0.585
Diabetes mellitus	46 (18.3%)	10 (21.3%)	0.635
Hypertension	143 (57.0%)	20 (42.6%)	0.068
Atrial fibrillation	80 (31.9%)	13 (27.7%)	0.567
Coronary artery disease	61 (24.3%)	12 (25.5%)	0.857
Previous TIA/ischemic stroke	40 (15.9%)	9 (19.1%)	0.586
Previous SDH	8 (3.2%)	4 (8.5%)	0.088
HAS-BLED score ≥ 3	55 (21.9%)	10 (21.3%)	0.923
CHA_2_DS_2_-VASc score ≥ 2	199 (79.3%)	38 (80.9%)	0.807
Antithrombotic therapy	136 (54.2%)	26 (55.3%)	0.886
Antiplatelet medication	59 (23.5%)	13 (27.7%)	0.542
Anticoagulant medication	88 (35.1%)	13 (27.7%)	0.325
Non-traumatic etiology	76 (30.3%)	21 (44.7%)	0.053
Preceding ASDH	55 (21.9%)	5 (10.6%)	0.077
MGS on admission (mean ± SD)	1.4 ± 0.8	1.3 ± 0.8	0.413
GCS on admission (mean ± SD)	14.3 ± 1.6	14.4 ± 1.4	0.478
Bilateral hematoma	78 (31.1%)	22 (46.8%)	0.036^*^
Hematoma diameter ≥ 20 mm	138 (55.9%)	32 (71.1%)	0.057
Max. hematoma diameter (mm, mean ± SD)	20.3 ± 6.0	21.9 ± 6.6	0.106
Midline shift (mm, mean ± SD)	7.4 ± 4.7	6.4 ± 4.8	0.187
Mixed hematoma density	92 (37.1%)	13 (28.9%)	0.291
Bilateral BHC for bilateral CSDH	29 (of 78, 37.2%)	8 (of 22, 36.4%)	0.944
Residual hematoma diameter (mm, mean ± SD)	8.7 ± 5.2	15.3 ± 8.5	< 0.001^*^
Postoperative TIA/ischemic stroke, AMI or DVT/PE	14 (5.7%) missing = 12	3 (6.8%)	0.730
Time until resumption of ATT (days, mean ± SD)	59.7 ± 36.4 missing = 39	122.0 ± 97.0 missing = 5	0.009^*^
No ATT resumption	19 (of 136, 14.0%)	2 (of 26, 7.7%)	0.532
Preoperative mRS 0–2	184 (73.9%) missing = 2	35 (81.4%) missing = 4	0.294
Postoperative mRS 0–2	167 (67.6%) missing = 4	33 (76.7%) missing = 4	0.232
3-year mortality	73 (29.1%)	10 (21.3%)	0.273

*ASDH* acute subdural hematoma, *TIA* transient ischemic attack, *MGS* Markwalder Grading Scale, *GCS* Glasgow Coma Scale, *BHC* burr-hole craniostomy, *AMI* acute myocardial infarction, *DVT/PE* deep vein thrombosis/pulmonary embolism, *mRS* modified Rankin Scale

Male gender and bilateral hematoma showed statistically significant differences between the recurrence and non-recurrence groups. Furthermore, in a logistic regression analysis bilateral hematoma and male gender independently predicted recurrence (Table 2).

**Table 2:** Results of a bivariate logistic regression analysis for potential independent predictors of recurrent CSDH

Variable	Adjusted odds ratio	95% confidence interval	*p* value
Male gender	2.363	1.089–5.128	0.030^*^
Bilateral hematoma	1.918	1.013–3.630	0.045^*^

**Fig. 2:**
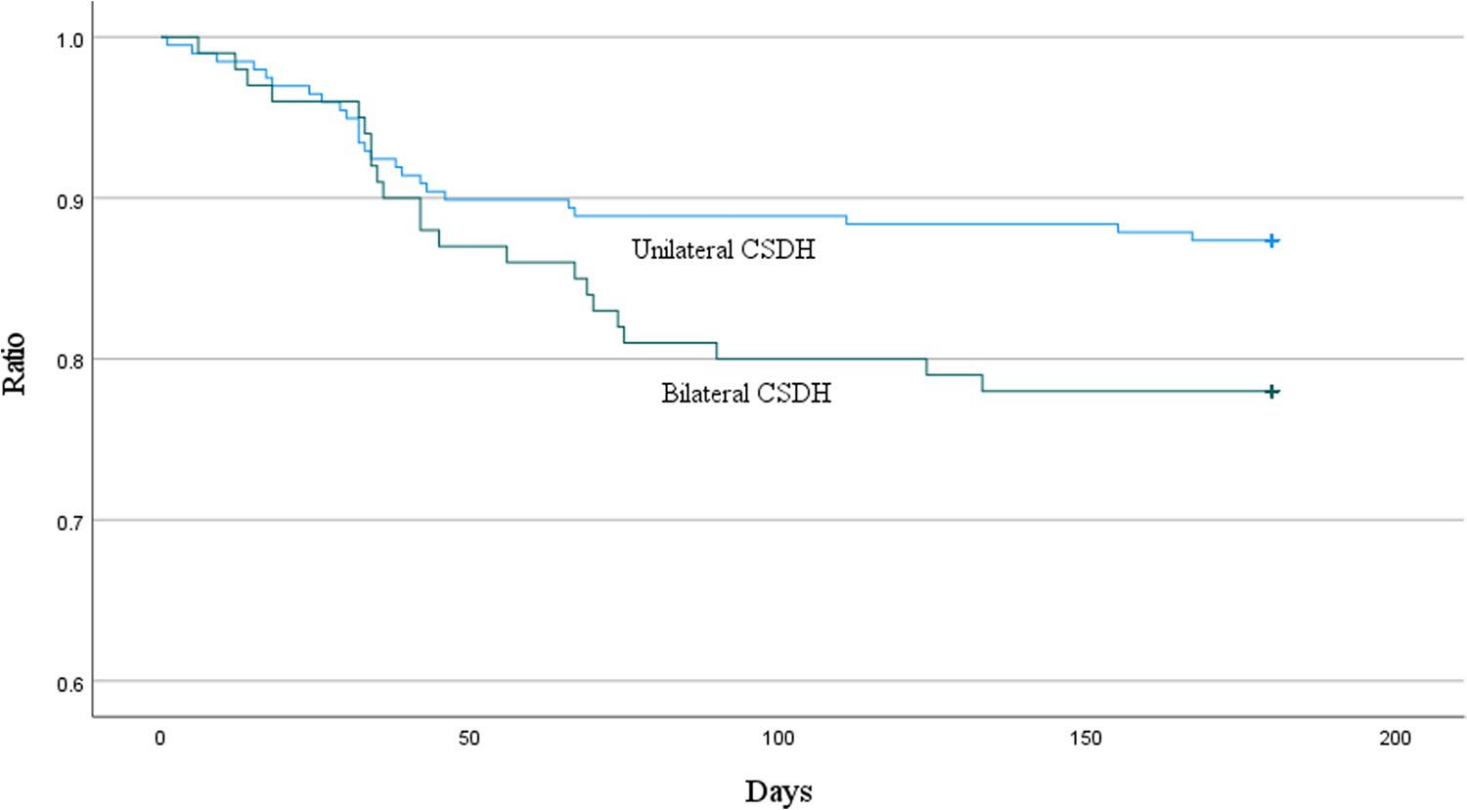
A Kaplan–Meier survival graph demonstrating the difference in 6-month recurrence risk in unilateral and bilateral CSDH. (IBM SPSS Statistics software (version 27.0.1.0) was used to create this figure)

**Fig. 3:**
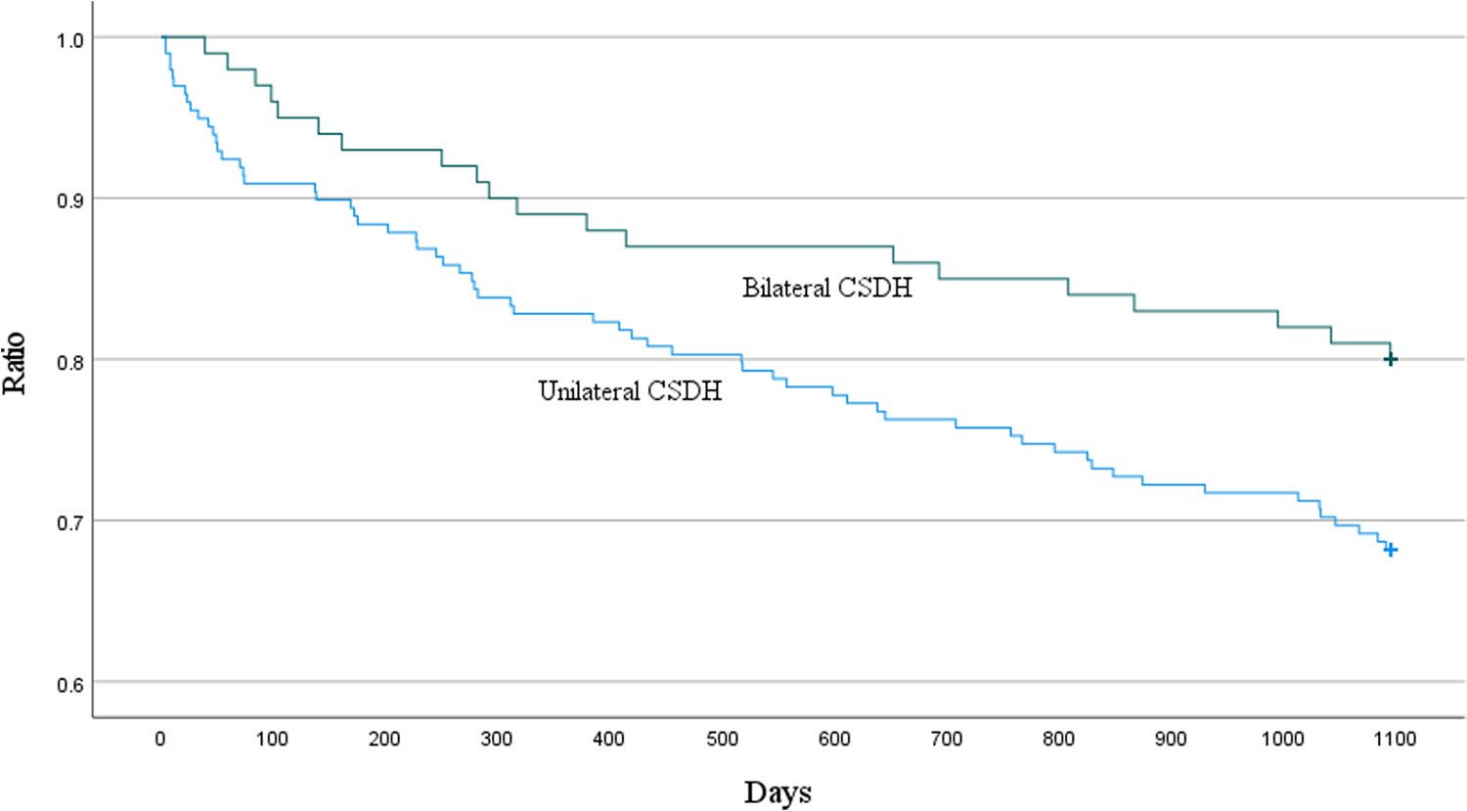
A Kaplan–Meier survival graph demonstrating the difference in 3-year survival in patients with unilateral and bilateral CSDH. (IBM SPSS Statistics software (version 27.0.1.0) was used to create this figure)

The characteristics of patients diagnosed with either unilateral or bilateral chronic subdural hematoma (bCSDH) (*n* = 199 and *n* = 102, respectively) were compared (Table 3). The recurrence rate was higher in the bCSDH group (12.6% vs. 22.0%; *p* = 0.036) (see Table 3 and Fig. 2), but the proportion of patients requiring more than one reoperation did not differ. Patients’ comorbidity profiles did not differ between the groups. The outcomes were worse for patients with unilateral hematomas in terms of 3-year mortality (31.8% vs. 20.0%; *p* = 0.032) (see Table 3 and Fig. 3).

**Table 3:** Comparison of patients with unilateral and bilateral chronic subdural hematoma

	Unilateral CSDH *N* = 199	Bilateral CSDH *N* = 102	*p* value
Age (years, mean ± SD)	76.8 ± 10.9	76.3 ± 9.4	0.699
Gender (male, *N* (%))	129 (64.8%)	71 (69.6%)	0.405
Memory disorder	44 (22.1%)	23 (22.5%)	0.931
Diabetes mellitus	41 (20.6%)	16 (15.7%)	0.303
Hypertension	114 (57.3%)	50 (49.0%)	0.173
Atrial fibrillation	56 (28.1%)	38 (37.3%)	0.106
Coronary artery disease	52 (26.1%)	21 (20.6%)	0.288
Previous TIA/ischemic stroke	33 (16.6%)	17 (16.7%)	0.985
Previous SDH	6 (3.0%)	6 (5.9%)	0.231
HAS-BLED score ≥ 3	45 (22.6%)	20 (19.6%)	0.549
CHA_2_DS_2_-VASc score ≥ 2	155 (77.9%)	84 (82.4%)	0.365
Antithrombotic therapy	109 (54.8%)	55 (53.9%)	0.888
Non-traumatic etiology	62 (31.2%)	36 (35.3%)	0.468
Preceding ASDH	52 (26.1%)	9 (8.8%)	< 0.001^*^
MGS on admission (mean ± SD)	1.5 ± 0.8	1.3 ± 0.7	0.030^*^
GCS on admission (mean ± SD)	14.2 ± 1.8	14.5 ± 0.9	0.021^*^
Hematoma diameter ≥ 20 mm	116 (59.5%)	56 (56.0%)	0.565
Max. hematoma diameter (mm, mean ± SD)	20.6 ± 6.1	20.4 ± 6.2	0.844
Midline shift (mm, mean ± SD)	8.6 ± 4.4	4.6 ± 4.4	< 0.001^*^
Mixed hematoma density	66 (33.7%)	40 (40.0%)	0.283
Residual hematoma diameter (mm, mean ± SD)	9.5 ± 6.3	10.4 ± 6.6	0.279
Recurrence	25 (12.6%) missing = 1	22 (22.0%) missing = 2	0.036^*^
Time until reoperation (days, mean ± SD)	44.4 ± 41.7	51.9 ± 33.5	0.508
More than one reoperation	5 (of 25, 20.0%)	4 (of 22, 18.2%)	1.000
No ATT resumption	14 (of 109, 12.8%)	7 (of 55, 12.7%)	0.983
Postoperative TIA/ischemic stroke, AMI or DVT/PE	12 (6.3%) missing = 8	5 (5.1%) missing = 4	0.686
Preoperative mRS 0–2	140 (72.5%) missing = 6	79 (79.8%)	0.175
Postoperative mRS 0–2	130 (67.7%) missing = 7	70 (71.4%) missing = 4	0.517
3-year mortality	63 (31.8%) missing = 1	20 (20.0%) missing = 2	0.032^*^

*ASDH* acute subdural hematoma, *TIA* transient ischemic attack, *MGS* Markwalder Grading Scale, *GCS* Glasgow Coma Scale, *AMI* acute myocardial infarction, *DVT/PE* deep vein thrombosis/pulmonary embolism, *mRS* modified Rankin Scale

Seventeen patients (5.7%) experienced a postoperative thromboembolic complication (thirteen strokes/TIAs, two acute myocardial infarctions and three deep vein thrombosis/pulmonary embolisms). Patients on ATT due to previously diagnosed atrial fibrillation did not have an increased risk for postoperative stroke/TIA as compared to patients without a history of atrial fibrillation (6.6% vs. 3.5%; *p* = 0.240). However, the risk for postoperative stroke/TIA was increased for patients with previous stroke/TIA (2.5% vs. 15.2%; *p* = 0.001). Only one postoperative stroke/TIA occurred in the recurrence group, and this patient was not on ATT preoperatively. Six other types of postoperative complications were reported in seven patients: two acute subdural hematomas, one intracerebral hemorrhage, seven seizures, one wound infection, one meningitis and two pneumonias.

Of the 102 patients with bCSDH, 63 (61.8%) were primarily operated with unilateral BHC. The recurrence rate did not depend on the primary operation (uni- or bilateral BHC, 22.2% vs. 21.6%; *p* = 0.944), and the need of more than one reoperation was not higher in the unilateral BHC group (21.4% vs. 12.5%; *p* = 1.000) (Table 4). The mortality rate was higher in patients with bCSDH who were operated with bilateral BHC (37.8% vs. 9.5%; *p* < 0.001).

**Table 4:** Comparison of patients with bilateral subdural hematoma treated either by unilateral or bilateral burr-hole craniostomy

	Unilateral BHC *N* = 63	Bilateral BHC *N* = 39	*p* value
Age (years, mean ± SD)	75.8 ± 10.2	77.0 ± 7.9	0.541
Gender (male, *N* (%))	45 (71.4%)	26 (66.7%)	0.611
Memory disorder	13 (20.6%)	10 (25.6%)	0.557
Diabetes mellitus	8 (12.7%)	8 (20.5%)	0.292
Hypertension	35 (55.6%)	15 (38.5%)	0.093
Atrial fibrillation	21 (33.3%)	17 (43.6%)	0.298
Coronary artery disease	12 (19.0%)	9 (23.1%)	0.625
Previous TIA/ischemic stroke	8 (12.7%)	9 (23.1%)	0.172
Previous SDH	4 (6.3%)	2 (5.1%)	1.000
Antithrombotic therapy	34 (54.0%)	21 (53.8%)	0.990
HAS-BLED score ≥ 3	10 (15.9%)	10 (25.6%)	0.227
CHA_2_DS_2_-VASc score ≥ 2	52 (82.5%)	32 (82.1%)	0.950
Non-traumatic etiology	22 (34.9%)	14 (35.9%)	0.920
Preceding ASDH	5 (7.9%)	4 (10.3%)	0.729
MGS on admission (mean ± SD)	1.3 ± 0.7	1.3 ± 0.6	0.931
GCS on admission (mean ± SD)	14.5 ± 0.9	14.5 ± 1.1	0.783
Max. hematoma diameter (mm, mean ± SD)	20.8 ± 6.8	19.8 ± 5.2	0.466
Contralateral hematoma diameter (mm, mean ± SD)	9.2 ± 4.0	14.3 ± 3.9	< 0.001^*^
Midline shift (mm, mean ± SD)	6.2 ± 4.5	2.0 ± 2.6	< 0.001^*^
Mixed hematoma density	24 (38.7%)	16 (42.1%)	0.737
Residual hematoma diameter (mm, mean ± SD)	9.4 ± 5.9	12.1 ± 7.4	0.059
Contralateral residual hematoma diameter (mm, mean ± SD)	8.8 ± 5.9	7.4 ± 5.2	0.287
Recurrence	14 (22.2%)	8 (21.6%) missing = 2	0.944
More than one reoperation	3 (of 14, 21.4%)	1 (of 8, 12.5%)	1.000
Postoperative TIA/ischemic stroke, AMI or DVT/PE	1 (1.6%) missing = 1	4 (11.1%) missing = 3	0.059
Preoperative mRS 0–2	52 (83.9%) missing = 1	27 (73.0%) missing = 2	0.191
Postoperative mRS 0–2	48 (78.7%) missing = 2	22 (59.5%) missing = 2	0.041^*^
3-year mortality	6 (9.5%)	14 (37.8%) missing = 2	< 0.001^*^

*BHC* burr-hole craniostomy, *ASDH* acute subdural hematoma, *TIA* transient ischemic attack, *MGS* Markwalder Grading Scale, *GCS* Glasgow Coma Scale, *AMI* acute myocardial infarction, *DVT/PE* deep vein thrombosis/pulmonary embolism, *mRS* modified Rankin Scale

Three patients were lost from follow-up due to their permanent place of residence within the catchment area of another university hospital. For six patients the primary and for twenty patients, the follow-up radiological imaging data was insufficient. For forty-four patients, the indication for ATT was unclear, the use of ATT was sporadic or they died shortly after the operation. Hence, the ATT resumption time could not be reliably determined for these patients.

## Discussion

The recurrence rate of operated CSDHs in this population-based cohort was 15.8%, which is in line with previous studies reporting a recurrence risk from 6 to 29% [4, 8–10, 12–14, 17, 20, 21, 28, 41, 43, 44, 52, 54]. A regression analysis revealed that male gender and a bilateral hematoma are independent risk factors for recurrence, latter of which has been generally recognized also in previous literature [32]. Male gender has previously been reported to predispose to CSDH [53] and was associated with higher recurrence rate in a Danish population-based study in 2015 [42].

The role of antithrombotic therapy in CSDH recurrence is under debate. Several studies have shown ATT to be a risk factor for recurrence [12, 13, 18, 27, 44, 50], whereas a number of studies have come to the opposite conclusion [6, 14, 17, 22, 33, 45, 49]. In addition, different results have been reported between the effect of anticoagulant and antiplatelet medications. In a systematic review, it has been suggested that anticoagulant medication is associated with an increased recurrence risk, whereas antiplatelet medication is not [34]. In our study cohort, neither anticoagulant nor antiplatelet therapy was associated with an increased recurrence risk. The proportion of patients on ATT prior to surgery was 54.8%, which seems to be one of the highest rates reported.

Following surgery, the clinical decision making on ATT continuation is balanced individually between the risk of CSDH recurrence and the risk of thromboembolic complications [37]. The evidence on this issue is rather limited, and clinical guidelines do not exist. Previously, it has been demonstrated that the number of thromboembolic events tends to increase if the discontinuation of ATT is prolonged, and thus, generally prompt resumption is recommended [3, 17, 22]. However, no clear-cut evidence on the optimal duration of the length of the postoperative discontinuation exists [11, 35, 37]. In our study cohort, the ATT discontinuation was relatively long, but the results suggest that even long-term discontinuation may be safe (regardless of the indication of ATT) in terms of thromboembolic events, since their rate was similar in patients who did not experience hematoma recurrence (mean length of discontinuation 59.7 days) and in patients whose discontinuation was prolonged due to recurrence (mean length of discontinuation 122.0 days) (8.3% vs. 8.7%; *p* = 1.000). However, based on the current study conclusions cannot be drawn on whether a shorter ATT discontinuation period would be sufficient or whether a longer discontinuation would further reduce the recurrence risk. A multicenter randomized controlled trial is warranted to solve this issue.

The use of ATT did not differ between patients with unilateral and bilateral hematomas (Table 3) nor was there a difference in the subgroup of patients with bilateral hematomas, who were operated either unilaterally or bilaterally (Table 4). Previous studies focusing on bCSDH are strikingly limited considering the high frequency and the clinical significance of this condition. The clinical course of the disease may be milder than in unilateral cases, which possibly leads to a delay in the treatment [23]. On the other hand, rapid symptom aggravation can occur [1, 26]. The limited invasiveness of BHC for treating CSDH enables simultaneous evacuation of bilateral hematomas in most patients, although it is not known whether this leads to a better outcome as opposed to treating only the side with the larger hematoma. Bilateral (BHC) treatment was recommended by a Danish group in 2017 based on a hypothesis that the decrease in intracranial pressure caused by unilateral surgery would explain the growth of the contralateral unoperated hematoma [5]. In accordance with this, Zolfaghari and colleagues suggested to consider bilateral evacuation even if the diameter of the contralateral hematoma is below 15 mm [55].

Our data suggests that in bCSDHs, unilateral BHC would be the treatment method-of-choice if the diameter of contralateral hematoma is relatively small. In this study cohort, the mean diameter of the unoperated contralateral hematoma was 9.2 mm, which can be considered as a possible cutoff point in future studies. Furthermore, we argue that it is feasible to consider a unilateral surgical approach even in larger hematomas, since in our results the recurrence rates did not differ between the uni- and bilateral BHC subgroups, and the 3-year mortality appeared to be even higher among the bilaterally operated patients.

The annual incidence of CSDHs requiring surgical evacuation in this population-based cohort was 20.3/100 000 including all patient groups and etiologies. This is in line with recent reports from Finland, which have shown an increasing incidence of CSDHs over the last decades [38, 40, 48], and similar incidences have been reported also in other developed countries. [7, 24] This has been attributed to the widespread use of CT imaging, increased life expectancy and physical activity of elderly people as well as the use of antithrombotic medications. The increasing incidence of CSDHs leads to an increasing socio-economical burden and warrants future randomized studies to optimize treatment algorithms.

## Limitations

This is a retrospective register study which is predisposed to biases of observational research. The indications for the use of ATT were partly unclear, and some patients lacked radiological imaging data, which reduced the number of patients in some statistical analyses. Reoperations for recurrent hematomas were performed only for symptomatic cases. However, the routine follow-up CT imaging may have led to reoperations in some cases with very mild clinical symptoms. The strength of this study lies within the population-based, non-selected cohort of consecutive patients, all of whom were treated in a single tertiary center and followed up with a similar protocol.

## Conclusions

In this population-based register study, we found that preoperative antithrombotic therapy did not increase the recurrence risk of chronic subdural hematomas. Male gender and bilateral hematomas were more frequently associated with recurrences.

## Abbreviations

SDH: Subdural hematoma
CSDH: Chronic subdural hematoma
bCSDH: Bilateral chronic subdural hematoma
ATT: Antithrombotic therapy
BHC: Burr-hole craniostomy
ASDH: Acute subdural hematoma
ICH: Intracerebral hemorrhage,
TIA: Transient ischemic attack
